# Relapsing Fever Group Borreliae in Human-Biting Soft Ticks, Brazil

**DOI:** 10.3201/eid2701.200349

**Published:** 2021-01

**Authors:** Sebastián Muñoz-Leal, Álvaro A. Faccini-Martínez, Bruno M. Teixeira, Maria Marlene Martins, Maria Carolina A. Serpa, Glauber M.B. Oliveira, Felipe R. Jorge, Richard C. Pacheco, Francisco B. Costa, Hermes R. Luz, Marcelo B. Labruna

**Affiliations:** Universidade de São Paulo, São Paulo, Brazil (S. Muñoz-Leal, M.C.A. Serpa, G.M.B. Oliveira, M.B. Labruna);; Asociación Colombiana de Infectología, Bogotá, Colombia (Á.A. Faccini-Martínez);; Centro Universitario INTA–UNINTA, Sobral, Brazil (B.M. Teixeira, F.R. Jorge);; Universidade Federal de Uberlândia, Uberlândia, Brazil (M.M. Martins);; Universidade Federal de Mato Grosso, Cuiabá, Brazil (R.C. Pacheco); Universidade Estadual do Maranhão, São Luís, Brazil (F.B. Costa);; Universidade Federal do Maranhão, São Luís (H.R. Luz)

**Keywords:** *Ornithodoros*, ticks, spirochetes, parasites, relapsing fever group borreliae, *Borrelia*, Argasidae, South America, Brazil

## Abstract

We conducted a molecular survey for *Borrelia* spp. in *Ornithodoros* ticks previously reported as biting humans. We collected specimens in natural ecosystems and inside human dwellings in 6 states in Brazil. Phylogenetic analyses unveiled the occurrence of 4 putatively new species of relapsing fever group borreliae.

Tick-borne relapsing fever (TBRF) is a vectorborne disease caused by spirochetes of the genus *Borrelia* that thrive in enzootic cycles and are transmitted mainly by soft ticks of the genus *Ornithodoros* (*1*). Humans bitten by infected ticks can become ill and present a typical recurrent febrile syndrome (*1*). In the New World, research on TBRF persists mainly in North America, where *Borrelia turicatae*, *B. parkeri*, and *B. hermsii* infect humans (*1*). Meanwhile, the knowledge on relapsing fever spirochetes in South America has remained comparatively incomplete. In Brazil, *Ornithodoros brasiliensis*, *O. fonsecai, O. mimon*, *O. rietcorreai*, and *O. rostratus* ticks have been reported to parasitize humans (*2*,*3*), yet their role as vectors of *Borrelia* spp. is unknown. Recently, in Brazil, *B. venezuelensis*, the agent of South American TBRF during the first half of the 20th century, was isolated from the anthropophilic tick *O. rudis* (*4*). This finding highlighted the occurrence of pathogenic relapsing fever group borreliae (RFGB) and called attention to study human-biting *Ornithodoros* ticks as possible vectors of these microorganisms.

During December 2018–October 2019, we conducted collections of soft ticks in the Brazilian states of Ceará (CE), Goiás (GO), Mato Grosso (MT), Mato Grosso do Sul (MS), Maranhão (MA), and Rondônia (RO) (Appendix Figure). Collections in MS were implemented using dry ice as an attractor; in CE, GO, MA, and RO, we collected soft ticks inside caves, abandoned nests or between rocks in rural areas. In MT, specimens were collected on the walls of an inhabited house in an urban area. Collections of ticks were authorized by Instituto Chico Mendes de Conservação da Biodiversidade (ICMBio permits 65137-1 and 36413-1). 

A total of 665 specimens (236 males, 145 females, 284 nymphs) belonging to 8 species of the genus *Ornithodoros* were submitted to individual or pooled DNA extractions (Appendix Table 1). We screened extractions with a *Borrelia-*specific real-time PCR with primers Bor16S3F and Bor16S3R and probe Bor16S3P, using 2 μL of genomic DNA, to amplify a fragment of the 16S rRNA gene (*5*). Samples with cycle threshold values <32 were tested with a battery of PCRs targeting the 16S rRNA and the *flab* and *glpQ* borrelial genes.

Four species of ticks were positive by *Borrelia-* specific real-time PCR. We generated sequences of *Borrelia* 16S rRNA, *flaB*, and *glpQ* genes for these specimens (Appendix Table 1). Two haplotypes of 16S rRNA gene were sequenced from each of the 2 positive *O. mimon* ticks, and the obtained sequences of *flaB* and *glpQ* were identical for both specimens. One haplotype for each gene was obtained for *O. hasei* and the *Ornithodoros* sp. ticks from CE, and only a 16S rDNA sequence was obtained from *O. rietcorreai* ticks (Appendix Table 2). With high support values, Bayesian phylogenetic analyses showed that the *Borrelia* spp. characterized from *O. mimon*, *O. rietcorreai*, and the *Ornithodoros* sp. ticks from CE form a monophyletic clade related to RFGB occurring in the Old World. In turn, the *Borrelia* sp. harbored by *O. hasei* ticks clustered within New World RFGB (Figure). These results add further evidence that Old and New World RFGB do not necessarily have defined geographic distributions but rather correspond to arbitrary groups.

**Figure:**
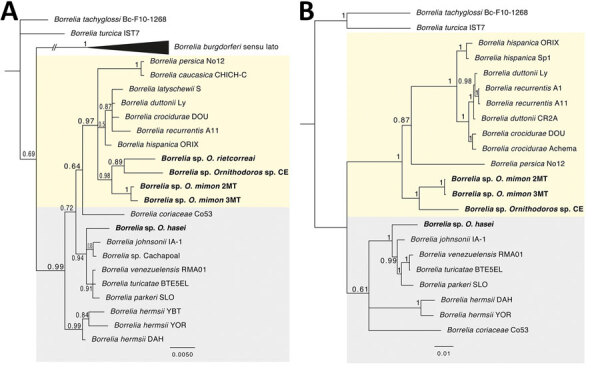
Bayesian phylogenetic trees inferred for the *Borrelia* spp. characterized in study of relapsing fever group borreliae in human-biting soft ticks, Brazil. A) Ambiguous alignments of single 16S rRNA gene (1,274 bp); B) concatenated 16S rRNA-*flaB-glpQ* genes (2,435 bp). Bold indicates borreliae from this study. Trees are drawn to scale. Four independent Markov chain runs for 1,000,000 metropolis-coupled MCMC generations were implemented for the analyses, sampling a tree every 100th generation. The first 25% of the trees represented burn-in, and the remaining trees were used to calculate Bayesian posterior probability values. Both trees were inferred using the Hasegawa-Kishino-Yano model with gamma distribution. Numbers above or below tree branches represent Bayesian posterior probabilities. Light yellow and gray backgrounds denote Old World and New World relapsing fever group *Borrelia* spp., respectively. Scale bar indicates nucleotide substitutions per site.

Five species of *Ornithodoros* ticks have been reported to parasitize humans in Brazil (*2*,*3*). We have added 2 more species to this list, as *O. hasei* and the *Ornithodoros* sp. ticks from CE avidly bit us during collections in the field (data not shown). Although with low prevalence, these 2 species, together with *O. mimon* and *O. rietocorreai*, harbored DNA of putatively new *Borrelia* spp. phylogenetically related to the relapsing fever group. The implications of these new spirochetes as human pathogens are still unknown. *O. mimon* and *O. rietcorreai* ticks are associated with human parasitism in urban and rural dwellings in Brazil (*2*,*3*), so vector roles of both species should not be overlooked.

TBRF courses with febrile episodes and should be considered as a differential diagnosis within the spectrum of diseases that cause an undifferentiated febrile syndrome (UFS) (*6*). Although specific data are vague for the states where tick collections were performed in this study, UFS is common in Brazil; mosquitoborne viruses and malaria are the main etiologic agents (*7*,*8*). Nevertheless, febrile illnesses still remain underdiagnosed in a substantial proportion of the cases in the country (*7*,*8*). The results of this study are a contribution to the knowledge of RFGB in human-biting *Ornithodoros* ticks, and stress the investigation of TBRF as a possible cause of UFS in Brazil. It is known that antibodies of patients exposed to RFGB infection cross-react in serologic tests for the diagnosis of Lyme borreliosis (*9*). This cross-reactivity is particularly relevant in Brazil because serologic evidence for an alleged Lyme-like disease in humans has been reiteratively published, yet refuted (*10*), and TBRF has not yet been considered as a possible cause of such disease.

Appendix Additional information on relapsing fever group borreliae in human-biting soft ticks.
