# Supplementary material for: Relapsing Fever Group Borreliae in Human-Biting Soft Ticks, Brazil
Source: Emerg Infect Dis. 2021 Jan;27(1):321–4. doi: 10.3201/eid2701.200349 (PMC7774541; doi:10.3201/eid2701.200349)
Supplement: Appendix — Additional information on relapsing fever group borreliae in human-biting soft ticks. [file 20-0349-Techapp-s1.pdf]

Article DOI: https://doi.org/10.3201/eid2701.200349

Relapsing Fever Group Borreliae in Human-Biting Soft Ticks, Brazil

Appendix

Supplemental Methods

Collected ticks were morphologically identified following original descriptions and redescriptions of South American species (*1*–*5*), and by comparisons with specimens deposited in the tick collection Coleção Nacional de Carrapatos Danilo Gonçalves Saraiva, São Paulo, Brazil. DNA extraction was performed using a phenol chloroform-based protocol (*6*). Successful extractions were confirmed by amplifying tick mitochondrial 16S rRNA gene with primers 16S+1 and 16S–1 in all the samples (*7*). Amplicons of this locus were sequenced only for soft ticks collected in new localities for the country, confirming their morphological identities by a phylogenetic analysis (data not shown). Amplicons of expected size for tick mitochondrial 16S rRNA gene and *Borrelia* 16S rRNA, *flab*, and *glpQ* genes were treated with Illustra ExoproStar (GE Healthcare, https://www.gehealthcare.com) and sequenced using an ABI 3500 genetic analyzer with a BigDye Terminator v3.1 Cycle Sequencing Kit (Applied Biosystems, https://www.thermofisher.com). Sequences were assembled and analyzed using Geneious R9 (*8*). Quality values (Q) for base calls were scaled between Q20 (error probability of 1 in 100) and Q40 (error probability of 1 in 10,000). Scores <Q20 in the 5′ and 3′ ends of each sequence were automatically trimmed. Values of quality, coverage, and length are shown in Appendix Table 2. BLASTn analyses were performed to infer most identical sequences in GenBank (https://blast.ncbi.nlm.nih.gov/Blast). Bayesian phylogenetic trees (http://mrbayes.csit.fsu.edu/) were inferred for single (*Borrelia* 16S rRNA) and concatenated (*Borrelia* 16S rRNA-*flaB*-*glpQ*) gene alignments constructed with MAFFT (https://mafft.cbrc.jp/alignment/server).

References

1. Labruna MB, Nava S, Marcili A, Barbieri ARM, Nunes PH, Horta MC, et al. A new argasid tick species (Acari: Argasidae) associated with the rock cavy, *Kerodon rupestris* Wied-Neuwied

(Rodentia: Caviidae), in a semiarid region of Brazil. Parasit Vectors. 2016;9:511. PubMed https://doi.org/10.1186/s13071-016-1796-7

2. Labruna MB, Nava S, Terassini FA, Onofrio VC, Barros-Battesti DM, Camargo LMA, et al. Description of adults and nymph, and redescription of the larva, of *Ornithodoros marinkellei* (Acari: Argasidae), with data on its phylogenetic position. J Parasitol. 2011;97:207–17. https://doi.org/10.1645/GE-2620.1

3. Labruna M, Venzal J. *Carios fonsecai* sp. nov. (Acari, Argasidae), a bat tick from the central-western region of Brazil. Acta Parasitol. 2009;54:355–63 https://doi.org/10.2478/s11686-009-0051-1

4. Barros-Battesti D, Onofrio V, Nieri-Bastos F, Soares J, Marcili A, Famadas K, et al. *Ornithodoros brasiliensis* Aragão (Acari: Argasidae): description of the larva, redescription of male and female, and neotype designation. Zootaxa. 2012;3178:22. https://doi.org/10.11646/zootaxa.3178.1.2

5. Cooley RA, Kohls GM. The Argasidae of North America, Central America and Cuba. The American Midland Naturalist Monograph No. 1. South Bend (IN): Notre Dame University Press; 1944. 152 p.

6. Sangioni LA, Horta MC, Vianna MCB, Gennari SM, Soares RM, Galvão MAM, et al. Rickettsial infection in animals and Brazilian spotted fever endemicity. Emerg Infect Dis. 2005;11:265–70. PubMed https://doi.org/10.3201/eid1102.040656

7. Mangold AJ, Bargues MD, Mas-Coma S. Mitochondrial 16S rDNA sequences and phylogenetic relationships of species of *Rhipicephalus* and other tick genera among Metastriata (Acari: Ixodidae). Parasitol Res. 1998;84:478–84. PubMed https://doi.org/10.1007/s004360050433

8. Kearse M, Moir R, Wilson A, Stones-Havas S, Cheung M, Sturrock S, et al. Geneious Basic: an integrated and extendable desktop software platform for the organization and analysis of sequence data. Bioinformatics. 2012;28:1647–9. PubMed https://doi.org/10.1093/bioinformatics/bts199

9. Schwan TG, Raffel SJ, Schrumpf ME, Policastro PF, Rawlings JA, Lane RS, et al. Phylogenetic analysis of the spirochetes *Borrelia parkeri* and *Borrelia turicatae* and the potential for tick-borne relapsing fever in Florida. J Clin Microbiol. 2005;43:3851–9. PubMed https://doi.org/10.1128/JCM.43.8.3851-3859.2005

10. Stromdahl EY, Williamson PC, Kollars TMJ Jr, Evans SR, Barry RK, Vince MA, et al. Evidence of *Borrelia lonestari* DNA in *Amblyomma americanum* (Acari: Ixodidae) removed from humans. J Clin Microbiol. 2003;41:5557–62. PubMed https://doi.org/10.1128/JCM.41.12.5557-5562.2003

Appendix Table 1. Results of molecular survey for *Borrelia* spp. in 8 *Ornithodoro*s species from different parts of Brazil

Species	Site of collection	Locality/state	No. tested specimens (N, M, F)	Pools/individual ticks	Real-time PCR positive samples (%)	GenBank accession no.,16S	GenBank accession no.,*flaB*	GenBank accession no.,*glpQ*
O. rudis	Abandoned bird nests in hollow palm trees	Araguapaz/GO	82 (15N, 35M, 32F)	Pools: 15N; 17M; 18M; 15F; 17F	0	None	None	None
*O. mimon**	Abandoned bird nests in hollow palm trees	Formosa da Serra Negra/MA	22 (11N, 7M, 4F)	Pools: 11N; 7M; 4F	0	None	None	None
	House	Cuiabá/MT	19 (8N, 9M, 2F)	Individual ticks	1F, 1N (10.5)	MT013211, MT013212	MT076262	MT076265
*O. hasei**	Cave	Jericoacoara National Park/CE	129 (48N, 51M, 20F)	Pools: 25N, 23N; 25M; 26M; 20F	23N pool (0.05†)	MT013210	MT076261	MT076264
*O. rietcorreai**	Between rocks	Serra das Almas Natural Reserve/CE	111 (75N, 23M, 13F)	Pools: 40N; 35N; 23M; 13F	13F pool (0.1†)	MT013213	None	None
Ornithodoros sp.*	Between rocks	Serra das Almas Natural Reserve/CE	39 (6N, 27M, 6F)	Pools: 6N; 27M; 6F	6F pool (0.2†)	MT013214	MT076263	MT076266
O. rostratus	Sandy soil	Fazenda São José/MS	171 (121N, 40M, 10F)	Individual ticks	0	None	None	None
O. marinkellei	Cave	Porto Velho/RO	68 (22M, 46F)	Individual ticks	0	None	None	None
*O. fonsecai**	Cave	Furna do Araticum/CE	34 (22M, 12F)	Individual ticks	0	None	None	None

*Tick with mitochondrial 16S rDNA sequence generated in this study (GenBank accession nos. MT021429, MT021430, MT021431, MT021432, MT021433, MT021434, MT021435). F, female; M, male; N, nymph.
†Minimal infection rate.
The following primers (forward/reverse) were used for PCR (‡) and sequencing (§) *Borrelia* genes: 16S rRNA gene: FD3/T50‡§, 16s-1/16s-2§, Rec4/Rec9§ (*9*); *flaB* gene: FLA LL/FLA LS‡§ (*10*); *glpQ* gene: glpQ F+1/ Rev-2‡§, Rev-1/2glpQ F-1§ (*9*).

Appendix Table 2. Coverage (mean ± standard deviation), quality values (% of base calls matching Q20, Q30, and Q40 values, discounting trimmed bases), and length (base pairs, bp) for the sequences of *Borrelia* 16S rRNA, *flab*, and *glpQ* genes obtained in this study

Tick species/*Borrelia* sp.	Gene	Coverage	Quality Q20	Quality Q30	Quality Q40	Trimmed length
Ornithodoros mimon						
Borrelia sp. Omi2MT	16S rRNA*	2.7 ± 0.8	92.9	90.6	87.6	1,342 bp
	flaB	5.5 ± 1.2	93.2	90.3	88.1	504 bp
	glpQ	3.3 ± 0.6	96.7	93.5	85.5	447 bp
Borrelia sp. Omi3MT	16S rRNA*	2.8 ± 0.8	94.1	92.4	89.1	1,342 bp
	flaB	5.5 ± 1.2	93.2	90.3	88.1	504 bp
	glpQ	3.3 ± 0.6	96.7	93.5	85.5	447 bp
Ornithodoros rietcorreai						
Borrelia sp. OrietCE	16S rRNA	2.8 ± 0.6	94.3	92.4	90.5	1,326 bp
Ornithodoros hasei						
Borrelia sp. JericoCE	16S rRNA	2.1 ± 0.9	95.5	90.8	85.9	1,345 bp
	flaB	3.1 ± 1.2	92.5	90.1	88.9	674 bp
	glpQ	2.9 ± 0.8	94.3	92.3	86.3	715 bp
Ornithodoros sp. CE						
Borrelia sp. Tabajara CE	16S rRNA					
	flaB	2.2 ± 1.1	98.6	96.8	95.5	614 bp
	glpQ	3.9 ± 0.8	96.4	94.7	92.4	598 bp

*Haplotypes for 16S rRNA gene of *Borrelia* sp. Omi2MT and *Borrelia* sp. Omi3MT differed in a single nucleotide with unambiguous base calls in each sequence (1341/1342 bp, 99.93% of identity).

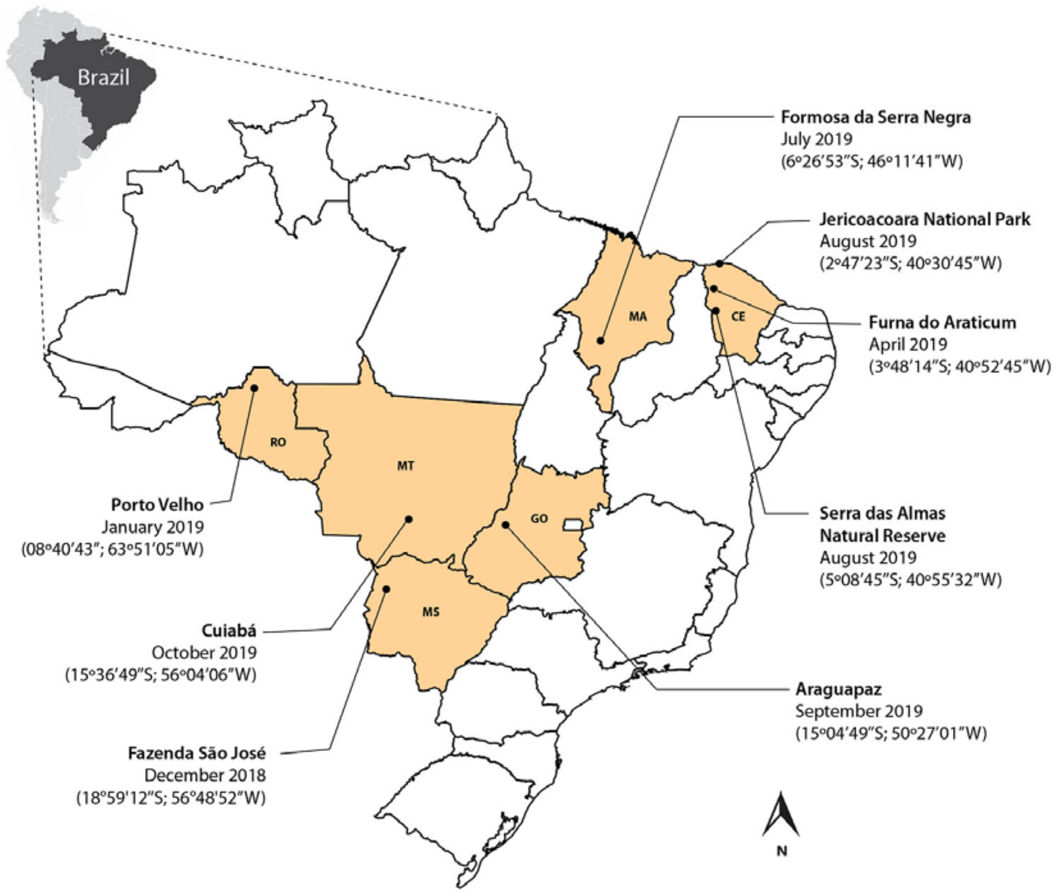

Appendix Figure. Map of Brazil showing the dates and localities where collection of ticks was performed. CE, Ceará State; GO, Goiás State; MA, Maranhão State; MS, Mato Grosso do Sul State; MT, Mato Grosso State; RO, Rondônia State.
